# Cryogenic nonlinear microscopy of high-Q metasurfaces coupled with transition metal dichalcogenide monolayers

**DOI:** 10.1515/nanoph-2024-0182

**Published:** 2024-06-06

**Authors:** Alena A. Nazarenko, Anna M. Chernyak, Alexander I. Musorin, Alexander S. Shorokhov, Lu Ding, Vytautas Valuckas, Milad Nonahal, Igor Aharonovich, Son Tung Ha, Arseniy I. Kuznetsov, Andrey A. Fedyanin

**Affiliations:** Faculty of Physics, 64935 Lomonosov Moscow State University, Moscow 119991, Russia; Department of Materials Science, Shenzhen MSU-BIT University, Shenzhen 517182, P.R. China; 54759 Institute of Materials Research and Engineering, A*STAR (Agency for Science, Technology and Research), Singapore, 138634, Singapore; School of Mathematical and Physical Sciences, Faculty of Science, 1994 University of Technology Sydney, Ultimo, New South Wales 2007, Australia; ARC Centre of Excellence for Transformative Meta-Optical Systems, 1994 University of Technology Sydney, Ultimo, New South Wales 2007, Australia

**Keywords:** transition metal dichalcogenides, monolayers, cryogenic temperature, nonlinear nanophotonics, all-dielectric metasurfaces, high-Q resonances

## Abstract

Monolayers of transition metal dichalcogenides (TMDCs) demonstrate plenty of unique properties due to the band structure. Symmetry breaking brings second-order susceptibility to meaningful values resulting in the enhancement of corresponding nonlinear effects. Cooling the TMDC films to cryogenic temperatures leads to the emergence of two distinct photoluminescence peaks caused by the exciton and trion formation. These intrinsic excitations are known to enhance second harmonic generation. The nonlinear signal can be greatly increased if these material resonances are boosted by high-quality factor geometric resonance of all-dielectric metasurfaces. Here, we experimentally observe optical second harmonic generation caused by excitons of 2D semiconductor MoSe_2_ at room and cryogenic temperatures enhanced by spectrally overlapped high-Q resonance of TiO_2_ nanodisks metasurface. The enhancement reaches two orders of magnitude compared to the case when the resonances are not spectrally overlapped.

## 1 Introduction

Nonlinear photonics gives a new breath to fundamental science and applications in data storage and information processing. Classical nonlinear optical effects require phase-matching fulfillment between fundamental and harmonic waves and are observed for prolongated anisotropic media or in materials with anomalous dispersion. Plasmonics and all-dielectric nanophotonics suggest a new approach to boost the effects through high local electromagnetic field confinement at a subwavelength area [1], [2]. Surface plasmon resonance in metals is known to enhance harmonic generation processes [3], [4], [5], [6], [7], [8], [9], [10]. However, light is concentrated only at the interface of the material and environment with weak penetration inside, limiting the efficiency of nonlinear conversion. On the contrary, high-index nonmetallic nano objects exhibit Mie scattering resonances and provide light–matter interaction within the entire volume of nonlinear material [11], [12], [13], [14], [15], [16], [17], [18], [19].

The improvement of fundamental radiation conversion to higher harmonics stimulates the exploration of new materials with high nonlinear susceptibility. 2D semiconductors attract more attention owing to the violation of inverse symmetry and the meaningful second-order susceptibility values [20], [21], [22]. Monolayers of transition metal dichalcogenides (TMDCs) demonstrate an absolute value of *χ* ^(2)^ of a few nm/V, which is much higher than that for classical nonlinear crystals [23], [24]. Mechanically exfoliated monolayer of MoS_2_ possesses a second-order nonlinear susceptibility that even reaches the order of 10^−7^ m/V [25], while its bulk counterpart exhibits the value of only 10^−14^ m/V [26]. Meanwhile, monolayers of MoSe_2_ possess nonlinear susceptibility of about 10^−11^ m/V [23]. Two-dimensional nature of TMDC usually forces to express a sheet second-order nonlinear susceptibility with the values of 10^−20^ m^2^/V [27], [28].

The symbiosis of TMDC with resonant structures proves to be a powerful way to enhance nonlinear conversion efficiency [7], [29], [30], [31]. Numerous papers focus on doubly resonant cavities [32], [33], [34]. The samples were designed to concentrate light outside the nanoresonators in the area of a TMDC monolayer, which is usually located at the top or bottom of the structure. Both strong light localization and the presence of appropriate electric field components oscillating in the plane of the monolayer are needed for harmonic generation. The enhancement up to 7,000 times is experimentally demonstrated in judiciously structured sub-20 nm-wide trenches on a 150 nm-thick gold film with WSe_2_ monolayer on top [7]. The design allows manipulating SHG intensity by rotating linear polarization of the pump laser. Patterned TMDC by itself can also enhance harmonic generation due to the high refractive index and excitation of Mie resonances [35], [36].

At helium temperatures (4–10 K), substances demonstrate unusual behavior governed by quantum rules. Some semiconductors become superconductive, while others, such as quantum dots or quantum wells, exhibit quantum effects that influence their optical properties – increased absorption and fluorescence, the appearance of higher-order excitons, etc. Coulomb interaction between one electron and one hole results in the generation of a neutral exciton (*X* ^0^), whose behavior is similar to a hydrogen atom. These quasiparticles can be transformed into the charged form of a three-particle exciton – trion, by binding to an additional electron (*X* ^−^) or an additional hole (*X* ^+^). The electronic and optical properties of such states are also of great importance for fundamental science and optoelectronic applications, ranging from emitting sources [37] to quantum logical devices [38], [39]. Both exciton and trion show a very sensitive dependence on temperature [40]. Measuring the photoluminescence (PL) spectrum is an easy way to track changes in the electronic configuration of MoSe_2_ films during the cooling process. It possesses one broadband peak at room temperature and two narrow peaks at cryogenic temperatures (15 K) corresponding to bright exciton and trion. These states become more pronounced at low temperatures. The PL spectrum line shape at 15 K differs for *X* ^0^ and *X* ^−^: the peak of the neutral exciton is symmetric and has homogeneous thermal broadening, while *X* ^−^ gives an asymmetric profile with a long low-energy tail consistent with electron-recoil effects. At temperatures above 55 K, the trion peak disappears since electrons leave the quasiparticle, which in turn occurs due to the increased influence of thermal fluctuations. This mechanism makes it possible to observe an increase in the value of nonlinear susceptibility for specific wavelengths, which leads to the enhancement of nonlinear optical signal compared to the room-temperature case. In the case of MoS_2_, experiments have shown the increasing time-integrated photoluminescence signal with cooling [41]. In contrast, for WSe_2_ monolayer, temperature-dependent PL decreases at the bright exciton peak and a 30-meV shifted dark state appearances during the cooling process. This different behavior is explained by the spin splitting of the conduction band, the sign of which differs for different TMDC materials.

**Figure 1:**
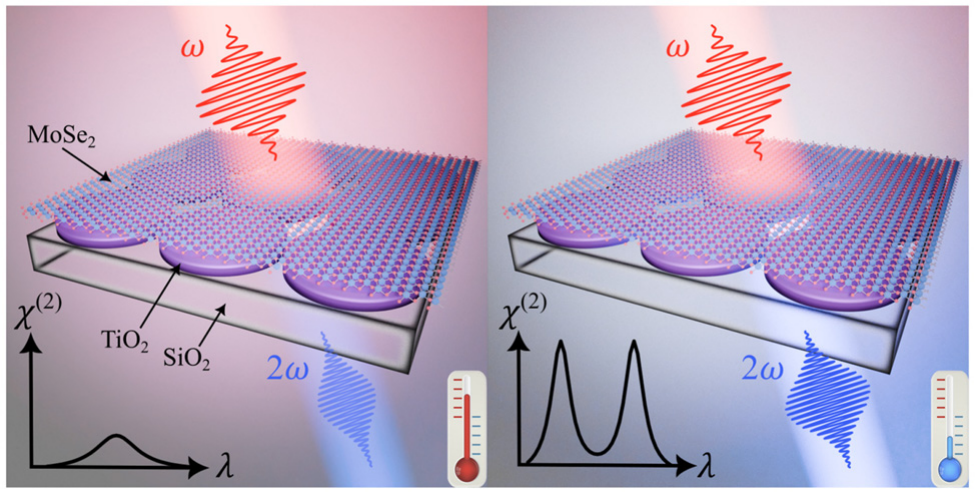
The idea of the experiment. Image to the left demonstrates second optical harmonic generation (SHG) in a MoSe_2_ monolayer coupled to the TiO_2_ metasurface at room temperature. This effect is governed by the high-Q geometric resonance of the nanostructure and a broad excitonic resonance of the 2D material. Image to the right demonstrates SHG boost caused by the interplay between the geometric resonance of the metasurface and narrowed material resonances of the MoSe_2_ monolayer under cryogenic temperature.

The spectral overlap of optical resonances of high-index nanocavities with excitonic states of TMDC monolayers leads to significant amplification of optical harmonic generation [30], [34], [42]. However, the interplay between cryogenic cooling and coupling of TMDC exciton with high-quality (high-Q) resonance of all-dielectric metasurfaces has not been studied yet. Here, we explore the nonlinear optical spectra of TiO_2_ metasurface covered by MoSe_2_ monolayer to elucidate this effect (see Figure 1).

## 2 Results and discussion

### 2.1 Linear spectroscopy

**Figure 2:**
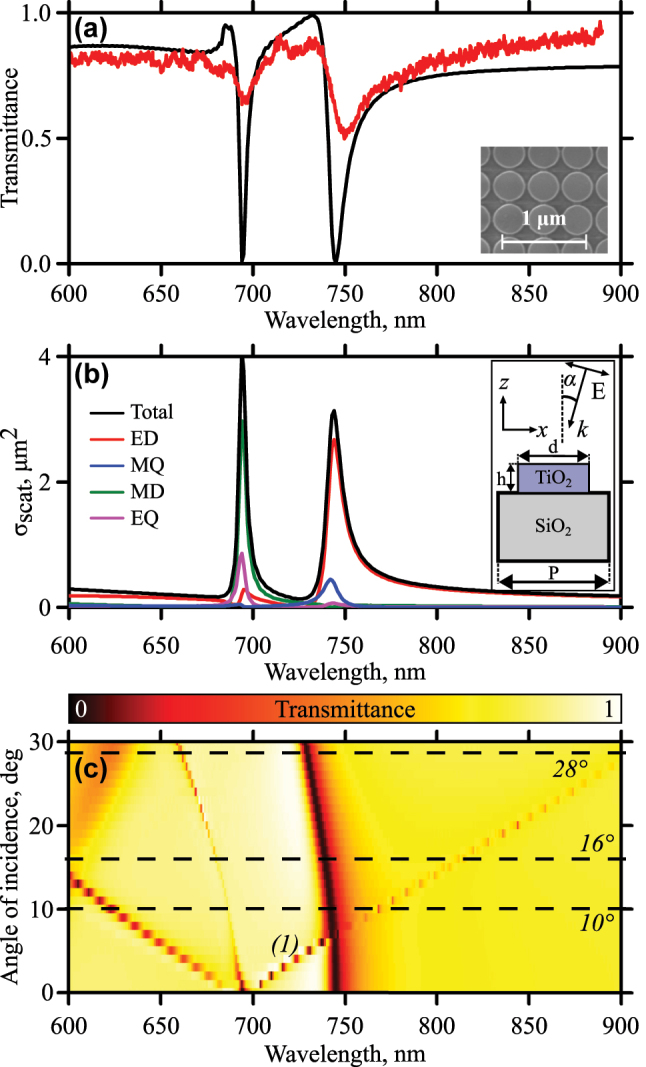
Linear optical characterization. (a) Transmittance spectra of the metasurface for normal incidence. The black curve is numerical calculations, red – experiment. The inset shows an SEM image of the sample. (b) Multipole decomposition. The inset shows the geometry of the sample and the experiment. (c) Angle-of-incidence-resolved numerical transmittance spectra of the sample. The dashed lines are angles chosen for nonlinear experiments.


As a resonant system, we study a dielectric metasurface that consists of a densely packed 2D array of TiO_2_ nanodisks on a transparent SiO_2_ substrate. The square lattice has the periods *P* of 470 nm. The cylinder diameter *d* is 450 nm and the height *h* is 120 nm. The size of the metasurface is 50 μm. The details on the sample fabrication can be found in the Supporting Materials (S1). The transmittance spectra of the sample for normal incidence are shown in Figure 2(a). The red curve is the experimental results and the black one is the numerical modeling. The inset shows a scanning electron microscopy (SEM) image of the metasurface. The spectral sensitivity of the linear experimental setup is limited by 900 nm and the scheme of the setup is presented in the Supporting Materials (S2.1). The details of numerical modeling can be found in the Supporting Materials as well (S3.1). The spectra have two resonant features. The nature of these resonances is the overlapped electrical dipole (ED, red curve) with magnetic quadrupole (MQ, blue curve) for the resonance at the wavelength of 745 nm and overlapped magnetic dipole (MD, green curve) with electric quadrupole (EQ, magenta curve) for the one at the wavelength of 695 nm according to the multipole decomposition shown in the Figure 2(b). The inset shows the geometry of the sample and the experiment. These resonances here and thereafter will be called geometric underlying in that they are not connected with the material resonances and properties of the media but are dictated by the nanostructuring only. The details of the decomposition can be found in the Supporting Materials (S3.2). The quality factor of the left resonance is about 185 and for the right one is approximately 80 extracted from the numerical data. The fitting formula for the resonances is the Fano formula [43]:

$$T={\left|a_{1}+ia_{2}+\frac{b}{\omega -\omega_{0}+i\gamma }\right|}^{2}.$$

Here, *ω*_0_ is the central frequency of the resonance, *γ* is the damping factor, and *a*_1_, *a*_2_, and *b* are real constants. The spectral positions of both resonances agree well for experimental and numerical data. The difference in depth of the dips is explained by deviations of the disk diameters in the array during the fabrication process averaging this parameter from the exact calculated one (evaluation gives ±10 nm) and finite convergence angle of incoming radiation in the optical experiment averaging the spectral width of the resonance (evaluation gives ±3°). The latter one limits the resolution of the spectra measurements. The numerical calculation also shows that the cooling process does not affect the transmittance spectrum significantly (see the Supporting Materials S3.3).

A comparison of field distribution for two polarizations (S and P) in the case of oblique incident radiation is carried out. The results are presented in the Supporting Materials (S3.4). In this situation, we are interested not only in large values of local fields in the structure but also in overlapping of these fields with nonlinear material. All radiation can be concentrated in the resonator and as a result make a small contribution to the generation of harmonics in the film. Thus, the localization of the field in the monolayer area is of prior importance when choosing the polarization of the incident wave. The strong field localization at the top of the cylinder is observed for P-polarized incoming light. In the case of S-polarized light, the field concentrates between the neighboring disks instead of their top. In addition, local field distributions for P-polarized light, obtained for SHG fields at *λ*/2, when excited at the fundamental wavelength *λ*, also exhibit local field concentration in the area of the flake (S3.7). All the above explains the necessity to use P-polarized light.

Figure 2(c) shows transmittance spectra for various angles of incidence of P-polarized incoming light. Variation of the angle of incidence from the normal case leads to shifts of the resonances. We are interested in the one that is marked on the graph as ‘(1).’ It moves toward longer wavelengths. Analyzing the behavior of diffraction maxima on the metasurface, we conclude that the described resonances are hybrid modes of the Mie resonance and the Rayleigh anomaly. The details of the analysis can be found in the Supporting Materials (S3.5). The shift of the resonance toward longer wavelengths allows the metasurface resonance to be spectrally overlapped with the resonances of the TMDC monolayer by tilting the sample. The MoSe_2_ exciton resonances are observed in vicinity of 800 nm [23]. However, the position of photoluminescence peak strongly depends on the temperature [40] and strain [44]. It is also sensitive to electrostatic potential. When transferring the film on top of different materials, strain value may be the same, but local electrostatic potential may behave differently. That is why, the constituent materials of the structure also play an important role in the position of the exciton resonance. TiO_2_ is not exactly a standard material for exfoliation. The results obtained for the monolayer exfoliated on TiO_2_ metasurface may differ from previously reported data for Si or SiO_2_ substrates. As for the results obtained in this article, three cases will be considered in the nonlinear experiments: 10° when resonances are not overlapped; 16° – overlapping the metasurface resonance with bright exciton of TMDC; 28° – overlapping of the metasurface resonance with the localized exciton of the MoSe_2_. These angles are marked in Figure 2(c) by the dash lines.

### 2.2 Nonlinear spectroscopy

The monolayer of MoSe_2_ is used as a nonlinear material, which has a high value of nonlinear susceptibility and an excitonic state around the wavelength of 800 nm. The flake is deterministically transferred onto the TiO_2_ metasurface using an aligned transfer technique (described in the Supporting Materials (S1)). Briefly, monocrystalline flakes are mechanically exfoliated onto PDMS film, and subsequently, monolayer flakes are identified using an optical microscope. The flakes are then picked up by a polymer stamp and positioned onto the nanostructure using dual 4-axis stages integrated with an optical microscope. This technique ensures micrometer-level accuracy in the pick-and-place process.

**Figure 3:**
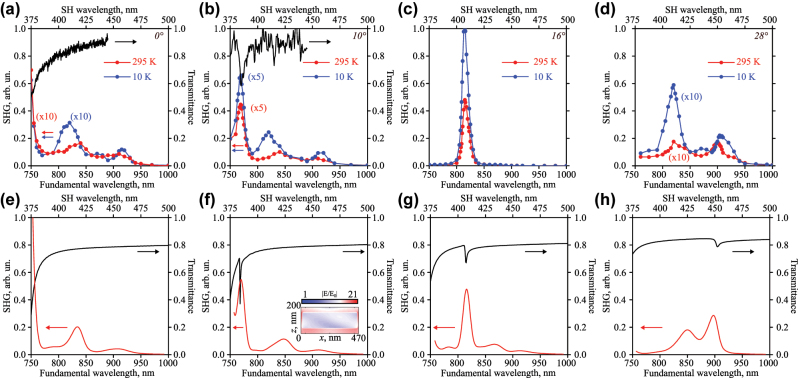
Optical second harmonic spectra for room temperature (red curves) and 10 K (blue curves), and transmittance spectra (black curves) for numerous angles of incidence: (a, e) 0°, (b, f) 10°, (c, g) 16°, (d, h) 28°. (a–d) Experiment, (e–h) numerical results. The inset on panel (f) shows normalized electric field distribution at the wavelength of the resonance of 770 nm in the plane of incidence *XOZ* in log scale.

Figure 3 shows both experimental and numerical results on second harmonic generation (SHG) spectroscopy. The nonlinear spectra are calculated only for the room temperature case by the finite element method in COMSOL Multiphysics software. The details of nonlinear calculations can be found in the Supporting Materials (S3.4). The measurements are performed at both room temperature (red dots) and 10 K (blue dots). All nonlinear spectra are normalized to the maximum SHG value observed for the case of overlapped geometric and excitonic resonances at 10 K. This value later will be denoted as $I_{2\omega }^{\max}$ . Such overlap occurs at the angle of incidence equal to 16° and at the wavelength of 815 nm (see Figure 3(c)). The details on the experimental setup can be found in the Supporting Materials (S2.2). Briefly, the source is a Ti:Sapp femtosecond oscillator with a repetition rate of 80 MHz, pulse duration of 150 fs tuned in the wavelength range of 680–1,080 nm. The mean power at the sample remains fixed at 100 mW for any fundamental wavelength. This limits the scanning range to wavelengths from 750 to 1,000 nm. The spot size at the sample plane is 200 μm, which means the fluence is about 4 μJ/cm^2^ and the peak intensity is about 0.03 GW/cm^2^. The detector is an EMCCD (electron-multiplying charge-coupled device) camera. The panels 3(a–d) represent measurement results, while the panels 3(e–h) depict numerical modeling. The black curves in Figure 3 are the transmittance spectra. The confirmation of quadratic dependence of SHG signal on the pump power is presented in the Supporting Materials (S2.3).

The geometric resonances are almost absent for normal incidence in the studied spectral region as discussed above in the linear spectroscopy section (see Figures 2 and 3(a, e)). One of the resonances is centered around 745 nm and its edge appears in Figure 3(a). The nonlinear spectrum at room temperature (see Figure 3(a), red dots) demonstrates enhancement in the vicinity of these wavelengths. Three other emerged resonant features are now associated with the band structure of the nonlinear 2D material. The nature of the left one (*λ* = 840 nm (1.47 eV)) is the bright exciton in MoSe_2_ [23]. The value of the SHG signal is only 1.6 % of $I_{2\omega }^{\max}$ . During the cooling process, its position shifts to the lower wavelengths (higher energies) up to 820 nm (1.51 eV) at 10 K. This effect is accompanied by resonance shrinkage and quality factor growth leading to a 2-fold enhancement of the second harmonic signal up to 3 % of the $I_{2\omega }^{\max}$ . This thermal behavior is typical and well-known from the literature [40]. Another interesting discovery resolved by second harmonic spectroscopy is the appearance of the trionic state of MoSe_2_ tightly connected to the cooling process and bright exciton shrinkage [45]. The corresponding peak is observed at the wavelength of 880 nm (1.41 eV), see Figure 3(a) blue dots. It is not very pronounced and has an amplitude of only 1 % of $I_{2\omega }^{\max}$ . The small binding energy of trions does not allow them to exist at room temperatures, so the peak is absent for room-temperature measurements. The final peak at the wavelength of 920 nm is associated with the localized exciton in MoSe_2_. It lies below the bright exciton and the trion (i.e., at higher wavelengths). The redshift of the bound exciton peak relative to the trion has already been demonstrated in SHG experiments [23]. The localized exciton peak is also visible at both low (*λ* = 920 nm) and room (*λ* = 905 nm) temperatures. This fact is consistent with the theory predicting two-dimensional TMDCs to be able to host a variety of bound exciton complexes, which are stable at room temperature [46], [47]. Its amplitude is much lower than that of the bright exciton [48]. It reaches about 1 % of the $I_{2\omega }^{\max}$ for both thermal cases. As for the origin of this state, traps can be related to defect potentials, which are often attributed to bound excitons in semiconductors [49], or can be related to the moiré potential caused by the presence of several monolayers in certain parts of the sample [50], [51]. In addition, the formation of localized states is observed even in monolayers [52]. This can be explained by the fact that the monolayer is not smooth enough, by its bending, by the presence of impurities, and the influence of the interface near the edge of the metasurface, where flakes bend toward the substrate. Previous studies of localized states in MoSe_2_ have revealed comb-like lines in photoluminescence spectra at energies below the trion state (with a redshift in wavelength) in certain areas of the flake. However, the exact prediction of the energy level diagram is still under discussion [53]. Similar effects are observed in WSe_2_ [54]. The exact nature of this resonance requires precise theoretical research, which is beyond the scope of this article as it focuses on nonlinear measurements. Moreover, following the previously obtained results, the position of the peak associated with the localized exciton slightly changes with the temperature, which is also consistent with literature [40].

The calculated nonlinear spectrum (see Figure 3(e)) at the room temperature case reproduces all main peculiarities: the enhancement of the signal for the geometric resonance near 750 nm, the signal for excitonic (*λ* = 840 nm) and localized (*λ* = 905 nm) resonances (compare red curves in the panels (a) and (e) in the Figure 3). The spectral positions are identical with the experiment, and the absolute values differ due to numerous factors that cannot be fully taken into account.

The minimum angle of incidence required to observe the geometric resonance for the studied wavelength range is 10° (see Figure 3(b, f)). The experimental (Figure 3(b)) and numerical (Figure 3(f)) transmittance spectra show the resonance near the wavelength of 770 nm. This high-Q feature arises from nearly equal contributions of the MD and EQ, with minor contributions from the ED and MQ multipoles according to the decomposition analysis (see the Supporting Materials (S3.2)). The SHG spectra demonstrate resonant amplification at both room and cryogenic temperatures. The spectral position does not change during cooling, which is expected, since this is dielectric-metasurface-induced resonance and there is no temperature dependence of TiO_2_ as mentioned above (Section 2.1 and Supporting Materials (S3.3)). The signal of the second harmonic at room temperature is 9 % of $I_{2\omega }^{\max}$ while at low temperature it increases up to 13 %, which is 1.5 times higher than that for the room temperature value. The SHG amplitudes for the room measurements remain unchanged and equal to 2 % for the bright exciton and 1 % for the localized exciton. The SHG values for low-temperature measurements are 5 % for the exciton and 2 % for the localized exciton. The numerical nonlinear spectrum (see Figure 3(f)) again reproduces all spectral features and their positions are in good agreement (compare red curves in the panels (b) and (f) in the Figure 3). The inset in the Figure 3(f) shows the local electric field distribution. The colormap represents the magnitude of the electric field sliced in the plane of incidence through the center of the disk for the unit cell of the metasurface at the wavelength of 770 nm. The hot spots are localized on the top edge of the disk and provide high field confinement inside the nonlinear 2D material, explaining the necessity to illuminate the sample with P-polarized light. These local fields yield significantly higher values of the nonlinear signal for the geometric resonance than the material resonances of MoSe_2_ give.

If the angle of incidence is set to 16°, the geometric resonance is spectrally overlapped with the material one of MoSe_2_ near the wavelength of 815 nm (see Figure 3(c, g)). The nonlinear signal increases by two orders of magnitude compared to the previously discussed results. The low-temperature signal of SHG is 2 times greater than that at room temperature. The nonlinear signal reaches the highest value of $I_{2\omega }^{\max}$ because of effective local field distribution, pumping the bright exciton of the TMDC film. The numerical transmittance spectra show a narrow and shallow dip (see Figure 3(g)). The experimental setup does not allow to resolve such peculiarities because of limited capabilities due to the reasons mentioned in the Section 2.1.

The next angle of incidence of 28° is chosen to overlap the geometric resonance with the localized excitonic state of MoSe_2_ near the wavelength of 900 nm (see Figure 3(d, h)). Nonlinear measurements (Figure 3(d)) show signal increase for the wavelengths of the bright (*λ* = 840 nm) and the localized (*λ* = 905 nm) excitons at room temperature. They reach 1.7 % $I_{2\omega }^{\max}$ for both cases, while these values differ (1.7 %@840 nm vs. 1 %@905 nm of $I_{2\omega }^{\max}$ ) for normal incidence when geometric and material resonances are not overlapped. It means that local electric field localization enhances nonlinear polarization. The numerical results (Figure 3(h), red curve) reproduce similar behavior. The black curve in Figure 3(h) demonstrates the transmittance spectrum with the weak resonance near the wavelength of 905 nm, which again cannot be resolved experimentally by the setup. The second harmonic signal increases by 3.5 times for the wavelength of the bright exciton (Figure 3(d), blue curve) and by 1.3 times at the wavelength of the localized exciton compared to the room-temperature case if the temperature decreases to 10 K. These values are 6 % of $I_{2\omega }^{\max}$ for the left spectral peculiarity and 2 % of $I_{2\omega }^{\max}$ for the right one. The enhancement for the localized exciton (*λ* = 920 nm) is less pronounced due to the temperature-dependent shift of the material resonance and misalignment with the position of the geometric resonance in the low-temperature case.

## 3 Conclusions

We experimentally observed the enhancement of optical second harmonic generation at cryogenic and room temperatures when material resonances of TMDC monolayers are spectrally overlapped with high-Q features of the all-dielectric metasurface. The SHG enhancement reaches two orders of magnitude compared to the case when the resonances are not at the same wavelength due to the interplay between the geometric and material resonances of the sample. The results can be inspiring for novel approaches to boost the development of optoelectronic science and nonlinear photonics.

## Supplementary Material

Supplementary Material Details

## References

[1] Yu N., Capasso F. (2014). Flat optics with designer metasurfaces. *Nat. Mater.*.

[2] Kuznetsov A. I., Miroshnichenko A. E., Brongersma M. L., Kivshar Y. S., Luk’yanchuk B. (2016). Optically resonant dielectric nanostructures. *Science*.

[3] Cai W., Vasudev A. P., Brongersma M. L. (2011). Electrically controlled nonlinear generation of light with plasmonics. *Science*.

[4] Celebrano M. (2015). Mode matching in multiresonant plasmonic nanoantennas for enhanced second harmonic generation. *Nat. Nanotechnol.*.

[5] Lippitz M., van Dijk M. A., Orrit M. (2005). Third-harmonic generation from single gold nanoparticles. *Nano Lett.*.

[6] Minovich A. (2012). Liquid crystal based nonlinear fishnet metamaterials. *Appl. Phys. Lett.*.

[7] Wang Z. (2018). Selectively plasmon-enhanced second-harmonic generation from monolayer tungsten diselenide on flexible substrates. *ACS Nano*.

[8] Afinogenov B. I., Popkova A. A., Bessonov V. O., Lukyanchuk B., Fedyanin A. A. (2018). Phase matching with Tamm plasmons for enhanced second-and third-harmonic generation. *Phys. Rev. B*.

[9] Aouani H., Rahmani M., Navarro-Cía M., Maier S. A. (2014). Third-harmonic-upconversion enhancement from a single semiconductor nanoparticle coupled to a plasmonic antenna. *Nat. Nanotechnol.*.

[10] Valev V. K. (2010). Asymmetric optical second-harmonic generation from chiral g-shaped gold nanostructures. *Phys. Rev. Lett.*.

[11] Shcherbakov M. R. (2014). Enhanced third-harmonic generation in silicon nanoparticles driven by magnetic response. *Nano Lett.*.

[12] Yang Y. (2015). Nonlinear fano-resonant dielectric metasurfaces. *Nano Lett.*.

[13] Kruk S. S. (2017). Nonlinear optical magnetism revealed by second-harmonic generation in nanoantennas. *Nano Lett.*.

[14] Li G., Zhang S., Zentgraf T. (2017). Nonlinear photonic metasurfaces. *Nat. Rev. Mater.*.

[15] Vabishchevich P. P., Liu S., Sinclair M. B., Keeler G. A., Peake G. M., Brener I. (2018). Enhanced second-harmonic generation using broken symmetry III–V semiconductor Fano metasurfaces. *ACS Photonics*.

[16] Camacho-Morales R. (2016). Nonlinear generation of vector beams from AlGaAs nanoantennas. *Nano Lett.*.

[17] Fedotova A. (2020). Second-harmonic generation in resonant nonlinear metasurfaces based on lithium niobate. *Nano Lett.*.

[18] Semmlinger M. (2019). Generating third harmonic vacuum ultraviolet light with a TiO_2_ metasurface. *Nano Lett.*.

[19] Carletti L., Kruk S. S., Bogdanov A. A., De Angelis C., Kivshar Y. (2019). High-harmonic generation at the nanoscale boosted by bound states in the continuum. *Phys. Rev. Res.*.

[20] You J. W., Bongu S. R., Bao Q., Panoiu N. C. (2018). Nonlinear optical properties and applications of 2D materials: theoretical and experimental aspects. *Nanophotonics*.

[21] Popkova A. A. (2021). Optical third-harmonic generation in hexagonal boron nitride thin films. *ACS Photonics*.

[22] Popkova A. A. (2022). Bloch surface wave-assisted ultrafast all-optical switching in graphene. *Adv. Opt. Mater.*.

[23] Le C. T. (2016). Nonlinear optical characteristics of monolayer MoSe_2_. *Ann. Phys.*.

[24] Choy M. M., Byer R. L. (1976). Accurate second-order susceptibility measurements of visible and infrared nonlinear crystals. *Phys. Rev. B*.

[25] Kumar N. (2013). Second harmonic microscopy of monolayer MoS_2_. *Phys. Rev. B*.

[26] Wagoner G. A., Persans P. D., Van Wagenen E. A., Korenowski G. M. (1998). Second-harmonic generation in molybdenum disulfide. *JOSA B*.

[27] Shen Y. R. (1989). Optical second harmonic generation at interfaces. *Annu. Rev. Phys. Chem.*.

[28] Lafeta L. (2021). Second-and third-order optical susceptibilities across excitons states in 2D monolayer transition metal dichalcogenides. *2D Mater.*.

[29] Day J. K., Chung M. H., Lee Y. H., Menon V. M. (2016). Microcavity enhanced second harmonic generation in 2D MoS_2_. *Opt. Mater. Express*.

[30] Löchner F. J. F. (2020). Hybrid dielectric metasurfaces for enhancing second-harmonic generation in chemical vapor deposition grown MoS_2_ monolayers. *ACS Photonics*.

[31] Chen H. (2017). Enhanced second-harmonic generation from two-dimensional MoSe_2_ on a silicon waveguide. *Light: Sci. Appl.*.

[32] Yi F. (2016). Optomechanical enhancement of doubly resonant 2D optical nonlinearity. *Nano Lett.*.

[33] Zanotti S., Minkov M., Fan S., Andreani L. C., Gerace D. (2021). Doubly-resonant photonic crystal cavities for efficient second-harmonic generation in III–V semiconductors. *Nanomaterials*.

[34] Hong P., Xu L., Rahmani M. (2022). Dual bound states in the continuum enhanced second harmonic generation with transition metal dichalcogenides monolayer. *Opto-Electron. Adv.*.

[35] Popkova A. A. (2022). Nonlinear exciton-Mie coupling in transition metal dichalcogenide nanoresonators. *Laser Photonics Rev.*.

[36] Busschaert S., Reimann R., Cavigelli M., Khelifa R., Jain A., Novotny L. (2020). Transition metal dichalcogenide resonators for second harmonic signal enhancement. *ACS Photonics*.

[37] Scholes G. D., Rumbles G. (2006). Excitons in nanoscale systems. *Nat. Mater.*.

[38] Stébé B., Ainane A. (1989). Ground state energy and optical absorption of excitonic trions in two dimensional semiconductors. *Superlattices Microstruct*..

[39] Kira M., Koch S. W., Smith R. P., Hunter A. E., Cundiff S. T. (2011). Quantum spectroscopy with Schrödinger-cat states. *Nat. Phys.*.

[40] Ross J. S. (2013). Electrical control of neutral and charged excitons in a monolayer semiconductor. *Nat. Commun.*.

[41] Zhang X. X., You Y., Zhao S. Y. F., Heinz T. F. (2015). Experimental evidence for dark excitons in monolayer WSe_2_. *Phys. Rev. Lett.*.

[42] Bernhardt N. (2020). Quasi-BIC resonant enhancement of second-harmonic generation in WS_2_ monolayers. *Nano Lett.*.

[43] Yang Y., Kravchenko I. I., Briggs D. P., Valentine J. (2014). All-dielectric metasurface analogue of electromagnetically induced transparency. *Nat. Commun.*.

[44] Liang J. (2017). Monitoring local strain vector in atomic-layered MoSe_2_ by second-harmonic generation. *Nano Lett.*.

[45] Zhou R., Krasnok A., Hussain N., Yang S., Ullah K. (2022). Controlling the harmonic generation in transition metal dichalcogenides and their heterostructures. *Nanophotonics*.

[46] Mostaani E. (2017). Diffusion quantum Monte Carlo study of excitonic complexes in two-dimensional transition-metal dichalcogenides. *Phys. Rev. B*.

[47] Jian-Jun L., Yu-xian L., Xiao-Jun K., Shu-Shen L. (1999). Binding energy of excitons bound to neutral donors in two-dimensional semiconductors. *Chin. Phys. Lett.*.

[48] Fang H. (2023). Localization and interaction of interlayer excitons in MoSe_2_/WSe_2_ heterobilayers. *Nat. Commun.*.

[49] De Greve K. (2010). Photon antibunching and magnetospectroscopy of a single fluorine donor in ZnSe. *Appl. Phys. Lett.*.

[50] Tran K. (2019). Evidence for moiré excitons in van der Waals heterostructures. *Nature*.

[51] Seyler K. L. (2019). Signatures of moiré-trapped valley excitons in MoSe_2_/WSe_2_ heterobilayers. *Nature*.

[52] Chakraborty C., Goodfellow K. M., Vamivakas A. N. (2016). Localized emission from defects in MoSe_2_ layers. *Opt. Mater. Express*.

[53] Jadczak J., Kutrowska-Girzycka J., Kapuściński P., Huang Y. S., Wójs A., Bryja L. (2017). Probing of free and localized excitons and trions in atomically thin WSe_2_, WS_2_, MoSe_2_ and MoS_2_ in photoluminescence and reflectivity experiments. *Nanotechnology*.

[54] Joshi J., Zhou T., Krylyuk S., Davydov A. V., Zutic I., Vora P. M. (2020). Localized excitons in NbSe_2_-MoSe_2_ heterostructures. *ACS Nano*.

