# Supplementary material for: Cryogenic nonlinear microscopy of high-Q metasurfaces coupled with transition metal dichalcogenide monolayers
Source: Nanophotonics. 2024 Jun 6;13(18):3429–36. doi: 10.1515/nanoph-2024-0182 (PMC11501421; doi:10.1515/nanoph-2024-0182)
Supplement: Supplementary file 1 — Supplementary Material Details [file j_nanoph-2024-0182_suppl_001.pdf]

Research Article

Alena A. Nazarenko, Anna M. Chernyak, Alexander I. Musorin, Alexander S. Shorokhov, Lu Ding, Vytautas Valuckas, Milad Nonahal, Igor Aharonovich, Son Tung Ha, Arseniy I. Kuznetsov, and Andrey A. Fedyanin*

Supplementary Material: Cryogenic non-linear microscopy of high-Q metasurfaces coupled with transition metal dichalcogenide monolayers

1 Sample fabrication

Substrates prepared for making TiO_2 metasurfaces were 120 nm-thick TiO_2 deposited by ion-assisted sputtering (Oxford Optofab3000) on the fused silica (quartz) substrates and followed by 30 nm-thick chromium (Cr) by electron-beam (e-beam) evaporation (Angstrom EvoVac). The deposited substrates were cleaned in deionized (DI) water, acetone, and isopropanol by ultra-sonication, and then performed dehydration bake at 120°C for 1 min. A negative e-beam resist, hydrogen silsesquioxane (HSQ, Dow Corning XR-1541-006) was spin-coated at 5000 rpm for 60 s, followed by a two-step baking at 120°C and 180°C for 2 min each. The metasurface arrays were patterned by e-beam lithography (Elionix ELS-7000) and HSQ were developed by a salty developer [1] (1 wt.% NaOH and 4 wt.% NaCl in DI water) for 4 min then rinsed with DI water. Using inductively coupled plasma reactive ion etching (ICP-RIE, Oxford PlasmaPro 100 Cobra), the Cr hard mask was etched, transferring the HSQ pattern, with a mixture of Cl_2 and O_2 gases. Then, the TiO_2 layer was etched with CHF_3 gas. Finally, Cr was removed by immersing the sample in liquid Cr etchant (Sigma Aldrich) for 15 min. The sample was rinsed by DI water and IPA and blow-dried with N_2 in the end.

Prior to the flake transfer, the wafer containing TiO_2 metasurface was meticulously cleaned to facilitate the transfer process. First, the wafer was sequentially rinsed with acetone and IPA followed by UVO cleaning for 30 minutes to remove organic residues. Subsequently, the wafer was annealed at 300 °C to enhance surface adhesion properties and remove any remaining contamination. The cleaning protocol is critical, as any residual impurities at the interface can significantly hinder the monolayer flake transfer process.

Monocrystalline $MoSe_2$ flakes were mechanically exfoliated from the bulk crystal onto PDMS film (supplied by Gel-Pak). Monolayer flakes were then identified under an optical microscope, distinguishable by their distinct optical contrast from few-layer stacks.

For the align transfer, the PDMS film bearing the monolayer $MoSe_2$ was mounted on an optical microscope equipped with dual 4-axis (x-y-z-θ) stages. One stage with a heating element supporting the wafer, while the other stage holds a polymer stamp. This arrangement enables precise manipulation of the flakes via pick-and-place technique.

The stamp was then drop-cast with a thin film of water-soluble polyvinyl alcohol (PVA), serving as a sacrificial layer to assist in the pickup and placement of the flakes. Afterwards, the selected flake was picked by the stamp and accurately positioned onto the target nanostructures. Subsequently, the PVA layer was thoroughly washed away using deionized (DI) water to ensure complete removal, leaving the flake intact on the

Alena A. Nazarenko, Anna M. Chernyak, Alexander I. Musorin, Alexander S. Shorokhov, Andrey A. Fedyanin, Faculty of Physics, Lomonosov Moscow State University, Moscow 119991, Russia, fedyanin@nanolab.phys.msu.ru
Alena A. Nazarenko, Anna M. Chernyak, Alexander I. Musorin, Department of Materials Science, Shenzhen MSU-BIT University, Shenzhen 517182, P.R.C.
Lu Ding, Vytautas Valuckas, Son Tung Ha, Arseniy I. Kuznetsov, Institute of Materials Research and Engineering, A*STAR (Agency for Science, Technology and Research), 138634, Singapore
Milad Nonahal, Igor Aharonovich, School of Mathematical and Physical Sciences, Faculty of Science, University of Technology Sydney, Ultimo, New South Wales 2007, Australia, Igor.Aharonovich@uts.edu.au
Milad Nonahal, Igor Aharonovich, ARC Centre of Excellence for Transformative Meta-Optical Systems, University of Technology Sydney, Ultimo, New South Wales 2007, Australia
Milad Nonahal, Current affiliation: Department of Chemistry, The University of Manchester, Oxford Road, Manchester, M13 9PL, UK, milad.nonahal@manchester.ac.uk

structures. Notably, throughout these steps, the stage heater was employed to adjust the stiffness of the PVA to facilitate the pick-and-place process.

2 Experimental setups

2.1 Linear measurements

The Figure 1 shows a scheme of the experimental

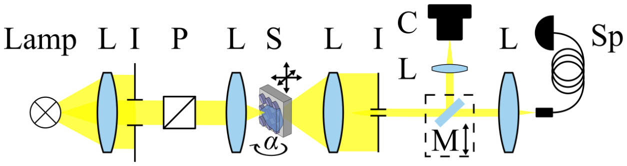

Fig. 1: Schematic illustration of the experimental setup for angle-resolved transmittance measurements. L — lenses, I — iris aperure, P — polarizer, S — sample, C — camera, M — mirror, Sp — spectrometer.

setup for angle-resolved transmittance spectra measurements. The radiation source is a halogen lamp with a wideband spectrum. It is placed in the focal plane of the lens to collimate the beam. The iris aperture allows one to control the radiation power. The light is polarized by a thin-film nanoparticle polarizer. Next, the light is focused on the sample by an aspherical lens. The sample can be rotated and can be positioned by a 3-axis stage. The radiation after the sample is collected by the lens. The second iris aperture allows the part of the light affected by the sample to pass through, and blocks the rest. This light field is focused into the fiber which is connected to the spectrometer. There is a movable auxiliary mirror that allows to steer the light into the visualization camera to control sample position relative to the beam.

2.2 Non-linear measurements

The experimental setup for optical second harmonic measurements in transmission geometry at the cryogenic temperature of 10K is shown schematically in the figure 2. The sample was mounted in a He-flow cryostat. The pump laser was a spectrally tunable femtosecond Ti:Sapphire laser with 150 fs pulse duration and repetition rate of 80 MHz. The wavelength of laser radiation was tuned in the range from 750 nm to 1000 nm. The radiation power and polarization were controlled

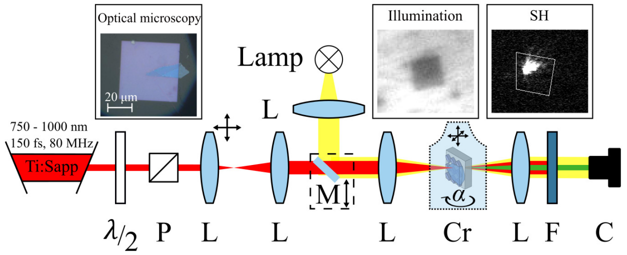

Fig. 2: Schematic illustration of the experimental setup for the observation of SHG in $MoSe_2$ monolayers coupled with metasurface of TiO_2 cylinders. The insets show an optical microscopy image of the sample (left), an example of the sample view by the EMCCD camera in the case of (middle) auxiliary illumination and (right) in the case of the second harmonic measurements. $\lambda/2$ — half-wave plate, P — polarizer, L — lenses, M — movable mirror, Cr — cryostate with the sample, F — set of blue filters, C — EMCCD camera.

by using a half-wave plate and a Glan prism. The power reaching the sample was kept constant (100 mW).

A telescopic system was introduced in the beam path to reduce the width of the collimated beam. The position of the incident beam spot on the sample was optimized by shifting one of the lenses in the telescopic system. Another telescope was used to focus the collimated beam on the sample.

The second harmonic signal was detected by the EMCCD camera. The signal on the camera was then integrated over the selected area corresponding to the monolayer coupled with the metasurface. To get rid of the fundamental radiation, a set of colored glass shortwave filters was placed before the camera. The visualization of the sample was made by an auxiliary channel with a broadband lamp and a beam splitter.

2.3 Power dependence of second harmonic signal

Figure 3 shows the log-log plot of the second harmonic power versus the pump power for the wavelength of 770 nm and the angle of incidence 10°. The dependence is fitted by the function $y = a + bx^c$. The power coefficient c of the fit is $c = 2.11 \pm 0.06$. The SHG power follows the expected quadratic dependence on the pump power.

Previous research shows that two-photon luminescence in $MoSe_2$ makes a small contribution at these wavelengths [2–4]. According to similar previous scientific papers devoted to the study of other TMDC materials (WS_2), it was shown that SHG signal strongly exceeds the multiphoton luminescence in the spectral

range we are studying, which is determined by a set of filters in front of the camera [5]. From all of the above, we conclude that we are dealing with second harmonic generation and the two-photon luminescence is negligible in the studied spectral range.

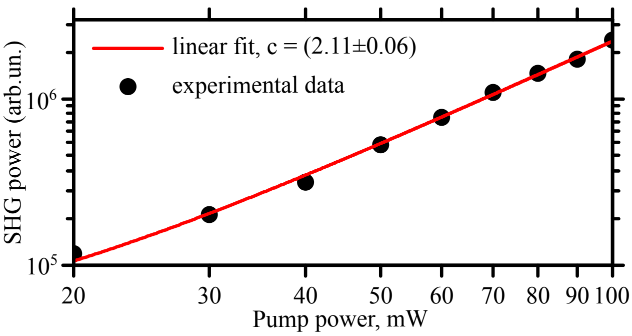

Fig. 3: Power-dependent nonlinear optical response plotted in double logarithmic scale. Black dots are experimental data and red curve is the approximation by power function $y = a + bx^c$, where $c = 2.11 \pm 0.06$.

3 Calculations

3.1 Linear spectroscopy

The linear angular-resolved transmittance spectra shown in Figure 2 of the main text were performed using the finite element method in COMSOL Multiphysics software. The frequency domain (Electromagnetic Waves, Frequency Domain) was used. The simulation area was constrained by perfectly matched layer domains in one dimension (z) and Floquet boundary conditions in the two remaining dimensions. Periodic Port boundary conditions were used for modeling plane waves incident upon the metasurface. Using these boundary conditions we specified the plane wave polarization and angle of incidence, taken in the range from 0° to 30°. The calculations were performed in the wavelength range from 600 nm to 900 nm.

3.2 Multipole decomposition

The nature of the resonances of the system under study was analyzed through the scattering cross-section of the four main fundamental Mie modes [6]. The equation for the impact of the modes on the total scattering cross-section is the following:

$$\sigma_{sc} \simeq \frac{k_0^4}{12\pi\varepsilon_0^2\mu_0 v_d I_0}\left|\mathbf{p} + \frac{ik_d}{v_d}\mathbf{T}\right|^2 + \frac{k_0^4\varepsilon_d}{12\pi\varepsilon_0 v_d I_0}|\mathbf{m}|^2 + $$

$$+\frac{k_0^6\varepsilon_d}{1440\pi\varepsilon_0^2\mu_0 v_d I_{inc}}\sum_{\alpha\beta}|Q_{\alpha\beta}|^2 + \frac{k_0^6\varepsilon_d^2}{160\pi\varepsilon_0 v_d I_0}\sum_{\alpha\beta}|M_{\alpha\beta}|^2 .$$

Here $\alpha = x, y, z$ and $\beta = x, y, z$; k_0 and k_d — wave

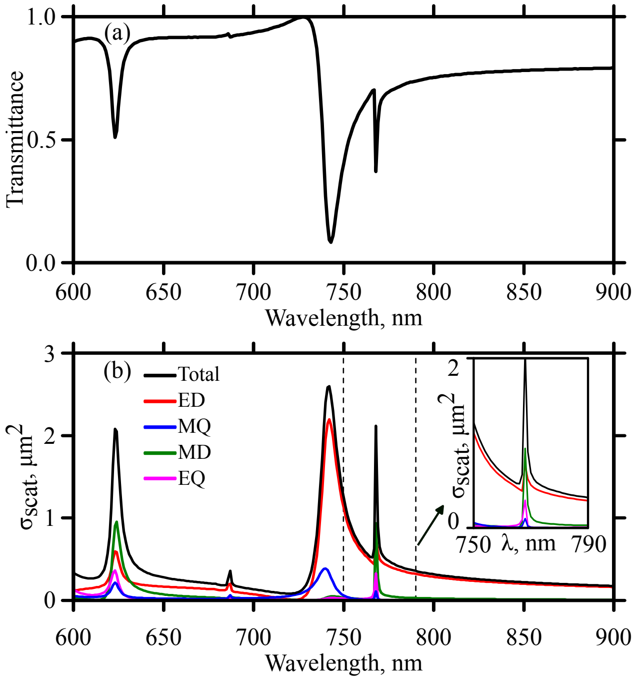

Fig. 4: (a) Transmittance spectra of the sample for P-polarized radiation incident at 10°. (b) Multipole decomposition. The inset is the zoom of the spectral region in the vicinity of the high-Q resonance.

vectors in the vacuum and in the medium with dielectric permeability ε_d; $v_d = c/\sqrt{\varepsilon_d}$ — light speed in the medium; ε_0, μ_0 — dielectric and magnetic permeability of the vacuum; I_0 — intensity of the incoming radiation; $\mathbf{p}$, $\mathbf{T}$ and $\mathbf{m}$ — electric (ED), toroidal (TD) and magnetic (MD) dipoles, $\hat{Q}$ and $\hat{M}$ — electric (EQ) and magnetic (MQ) quadrupoles.

The figure 4(a) shows the transmittance spectrum of the sample for 10° oblique incident P-polarized light. It has three main spectral features. The nature of these peculiarities is determined by the multipole decomposition shown in the figure 4(b). The most high-quality resonance at the wavelength of 770 nm is the combination of the MD mode and the slope of ED mode with a little influence of MQ and EQ. The middle resonance at 740 nm is mostly the ED mode. This feature almost

does not move with the increase of the angle of incidence. The last spectral dip at 625 nm is the sum of all four fundamental modes with MD dominant impact.

3.3 Temperature dependence of transmittance

The optical constants of $MoSe_2$ monolayer undergo changes during the cooling process from 300K to 10K [7], while the refractive index and absorption coefficient of TiO_2 are weakly dependent on the temperature [8]. Figure 5 shows the transmission spectrum

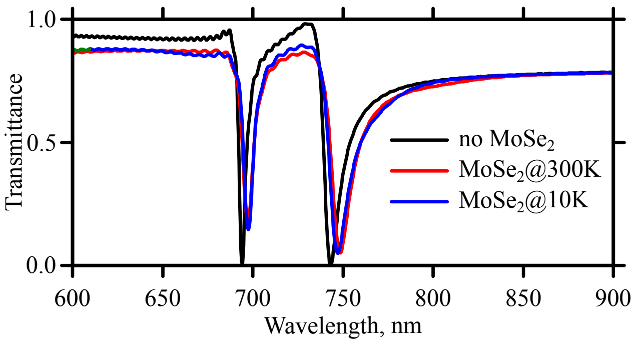

Fig. 5: Transmittance spectra of the metasurface for 3 cases: without $MoSe_2$ monolayer (black curve), with $MoSe_2$ monolayer at 300K (red curve) and 10K (blue curve).

of the metasurface without monolayer (black curve) and two spectra of the metasurface with the $MoSe_2$ monolayer at 300K and at 10K (red and blue curves respectively). The appearance of the 2D film manifests itself in a slight shift of the resonances due to variation on environment according to the Fresnel formulas. The quality factor of the resonance reduces a little bit. However, the cooling process does not affect significantly on the optical response due to the insignificant thickness. The calculations show minor changes.

3.4 Comparison of fields distribution for S and P polarizations

Figure 6 shows the transmittance spectra at the angle of incidence of 10° for the P- and S-polarized light (the panels (a) and (c) respectively) and the normalized electric field distribution in the case of P polarization at the wavelength of 770 nm (the panel (b)) and in the case of S polarization at the wavelength of 834 nm (the panel (d)). The cross-section is through the disk diameter for both cases. The hot spots for P-polarized light are at the top of the cylinder, while for S-polarized light the field concentrates between the neighboring disks. This explain the necessity of P-polarized light for non-linear measurements.

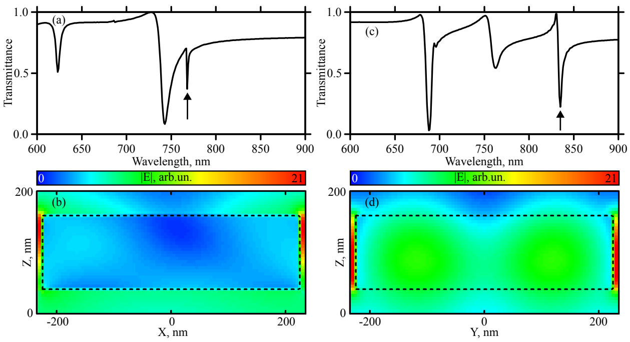

Fig. 6: (a) Transmittance spectrum for P-polarized light at angle of incidence 10°. The arrow shows the spectral position chosen for local electric field distribution plot. (b) Normalized local electric field distribution in the plane XOZ at the wavelength of 770 nm for P polarization. (c) Transmittance spectrum for S-polarized light at angle of incidence 10°. (d) Normalized local electric field distribution in the plane YOZ at the wavelength of 834 nm for S polarization.The arrow shows the spectral position chosen for local electric field distribution plot.

3.5 Diffraction

The Figure 7 describes the comparison of the transmittance spectra of the metasurface to the zero diffraction order with the total transmittance. The panel (a) is the same like in Figure 2(c) of the main text. The green curve in the panel (b) is the difference of the total and zero-order transmittance spectra for the angle of incidence of 10°. It shows that the leakage of the radiation power to the diffraction orders starts for the wavelength a little bit below the position of the geometric resonance. However, they are very close, so it would be correct to address this peculiarity as a hybrid mode. Finally, the panel (c) shows angle-resolved transmittance spectra to non-zero diffraction orders.

3.6 Non-linear spectroscopy

Numerical calculations of second harmonic spectra were also carried out using the finite element method in COMSOL Multiphysics software (Electromagnetic Waves, Frequency Domain). The Surface Current Den-

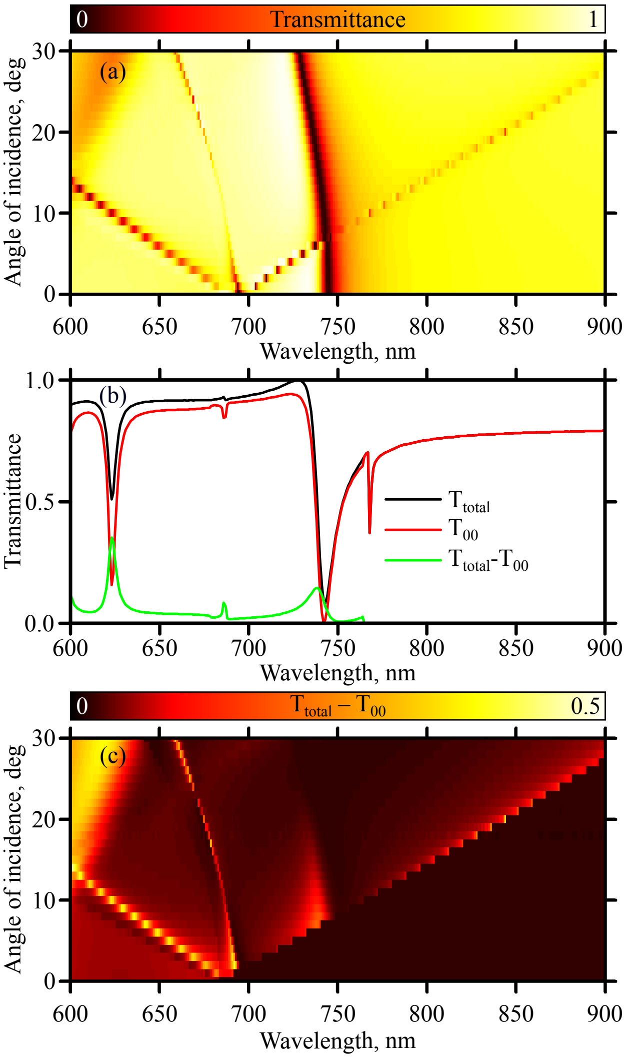

Fig. 7: (a) Angle-of-incidence-resolved numerical transmittance spectra of the sample. (b) Comparison of total transmittance spectra (black curve) with the transmittance to the (0,0) diffraction order (red curve) for 10° angle of incidence. The green curve shows the difference of these curves, i.e. transmittance to all non-zero diffraction orders. (c) Angle-resolved transmittance spectra to non-zero diffraction orders.

sity boundary condition was used to simulate the nonlinear properties of the $MoSe_2$ film. The relationship between surface current density, polarization, and the small thickness of the monolayer ($h_{\text{flake}} = 0.75$ nm) allows one to write the next equation:

$$J_s \approx \frac{\partial P}{\partial t} h_{\text{flake}}.$$

The second-order non-linear susceptibility spectrum was specified in the form of a Lorentz function (see Fig. 8). This dependence describes the two maxima of the $\chi^{(2)}$ value for the positions of exciton and bound state [9]. The parameters of the Lorentz function were

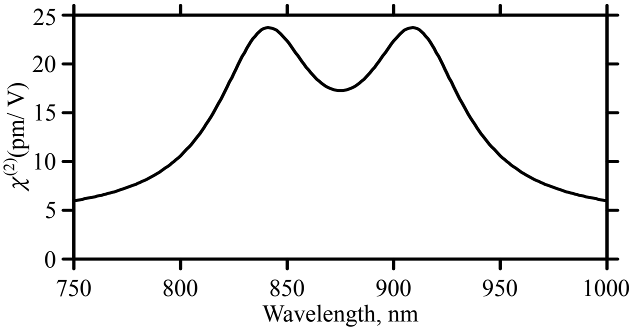

Fig. 8: Second-order susceptibility wavelength dependence.

chosen to achieve a good-quality correspondence with the experimental spectra. The magnitude of the susceptibility is similar to that one in previous study [9]. Knowing the nonlinear susceptibility, one can set the polarization of the domain in the following form:

$$P_x = \chi E_x E_x - \chi E_y E_y$$
$$P_y = -\chi E_y E_x - \chi E_x E_y$$
$$P_z = 0$$

These components underline the symmetric properties of the monolayer in the second-order nonlinear susceptibility tensor. The refractive index of the molybdenum diselenide monolayer was taken according to the literature [10].

3.7 SHG field distributions

Figure 9 shows the normalized electric field distribution in the case of P polarization in the plane XOZ.The cross-section is made through the center of the nanodisk. Figures (a), (b) refer to the case of radiation falling at an angle of 10°, (c), (d) refer to 16°. Figures (a), (c) illustrate the distribution of the fields at the fundamental wavelength, while (b), (d) reflect the distribution of SHG fields at a wavelength equal to half of the fundamental wavelength. The spectral positions of the metasurface resonances at a given angle of incidence act as the fundamental wavelength. It can be seen that hotspots at the top of the cylinder are observed both for the field distributions at the fundamental wavelength and for SHG field distributions. This configuration allows one to effectively pump a monolayer as a non-linear material, which will cover the metasurface, and, thus, obtain high values of SHG signal.

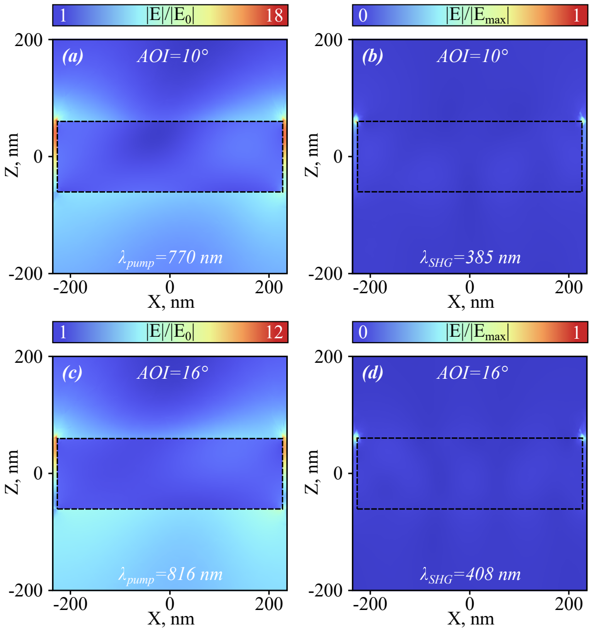

Fig. 9: Numerical results for normalized local electric field distributions in the plane XOZ for P-polarized excitation. (a) is the electric field distribution for the 770 nm excitation at 10° incidence; (b) corresponds to the SHG field distribution at a wavelength of 385 nm when excited by a fundamental wavelength of 770 nm at an angle of incidence equal to 10°; (c) the electric field distribution for the 816 nm excitation at 16° incidence; (d) SHG field distribution at 408 nm when excited by a fundamental wavelength of 816 nm at 16°. (a), (c) are normalized by the magnitude of the incident field, (b), (d) are normalized to the maximum value of the local field.

References

[1] J. K. W. Yang, K.K. Berggren, "Using high-contrast salty development of hydrogen silsesquioxane for sub-10-nm half-pitch lithography", *J. Vac. Sci. Technol. B* vol. 25, p. 2025–2029, 2007, https://doi.org/10.1116/1.2801881

[2] G. Wang, et al. "Exciton states in monolayer $MoSe_2$: impact on interband transitions", *2D Materials*, vol. 2, no. 4, p. 045005, 2015, https://doi.org/10.1088/2053-1583/2/4/045005

[3] K. Wang, et al., "Broadband ultrafast nonlinear absorption and nonlinear refraction of layered molybdenum dichalcogenide semiconductors", *Nanoscale*, vol. 6, no. 18, pp. 10530–10535, 2014, https://doi.org/10.1039/C4NR02634A

[4] R. Sharma, et al., "Strong interlayer coupling mediated giant two-photon absorption in $MoSe_2$/graphene oxide heterostructure: Quenching of exciton bands", *Phys. Rev. B*, vol. 93, no. 15, p. 155433, 2016, https://doi.org/10.1103/PhysRevB.93.155433

[5] S, Shiau, W. Li, Y, Chang, K. Lin, "Three-photon luminescence assisted by excitonic energy transfer in gold nanoparticle-WS_2 monolayers", *Cell Rep. Phys. Science*, vol. 4, no. 6, p. 101431, 2023, https://doi.org/10.1016/j.xcrp.2023.101431

[6] A. B. Evlyukhin, and C. Reinhardt, and B. N. Chichkov, "Multipole light scattering by nonspherical nanoparticles in the discrete dipole approximation", *Rhys. Rev. B*, vol. 84, no. 23, p. 235429, 2011, https://doi.org/10.1103/PhysRevB.84.235429

[7] H.L. Liu, et al. "Temperature-dependent optical constants of monolayer MoS_2, $MoSe_2$, WS_2, and WSe_2: spectroscopic ellipsometry and first-principles calculations", *Scientific reports*, vol. 10, no. 1, p. 15282, 2020 https://doi.org/10.1038/s41598-020-71808-y

[8] M. Isik, S. Delice, N. Gasanly, "Temperature-dependent optical properties of TiO_2 nanoparticles: a study of band gap evolution", *Opt. Quant. Electron.* vol. 55, p. 905, 2023, https://doi.org/10.1007/s11082-023-05138-4

[9] Le C. T. et al. "Nonlinear optical characteristics of monolayer $MoSe_2$", *Ann. Phys.*, vol. 528, pp. 551-559, 2016, https://doi.org/10.1002/andp.201600006

[10] C. Hsu, R. Frisenda, R. Schmidt, et al. "Thickness-dependent refractive index of 1L, 2L, and 3L MoS_2, $MoSe_2$, WS_2, and WSe_2", *Adv. Opt. Mater.*, vol. 7, p. 1900239, 2019, https://doi.org/10.1002/adom.201900239
